# Differences in Manifestations and Gut Microbiota Composition Between Patients With Different Henoch-Schonlein Purpura Phenotypes

**DOI:** 10.3389/fcimb.2021.641997

**Published:** 2021-07-01

**Authors:** Yuanzhen Zhang, Guizhi Xia, Xiaojing Nie, Yugui Zeng, Yi Chen, Yifang Qian, Guangming Chen, Jun Huang, Chengfeng Wang, Chuanyin Zhang, Xiaoli Huang, Yuen Yang, Xiaojian Qiu, Fang Yang, Jie Chen, Jun Hu

**Affiliations:** ^1^ Department of Pediatrics, Affiliated Dongfang Hospital, Xiamen University, Fuzhou, China; ^2^ Department of Pediatrics, The 900th Hospital of Joint Logistic Support Force, Fuzhou, China; ^3^ Department of Pediatrics, Fuzong Clinical Medical College of Fujian Medical University, Fuzhou, China; ^4^ Department of Pediatrics, The Fujian Provincial Maternity and Children’s Hospital, Fuzhou, China; ^5^ Department of Pediatrics, Fujian Provincial Hospital, Fuzhou, China; ^6^ Department of Pediatrics, Fujian Medical University Union Hospital, Fuzhou, China

**Keywords:** Henoch-Schonlein Purpura, manifestations, phenotypes, gut microbiota, autoimmune disease

## Abstract

**Background:**

Gut microbiota plays an important role in the pathogenesis of immune-mediated diseases. However, the complex pathogenesis of Henoch-Schonlein Purpura (HSP) remains elusive. This study aimed to characterize the gut microbiota in HSP patients and explore the potential association between gut microbiota composition and phenotypic changes in HSP.

**Methods:**

16SrRNA gene sequencing and bioinformatic analyses were performed using total DNA extracted from the fecal microbiota of 34 children with HSP, including 18 primary cases, 16 recurrent cases, and 23 healthy children.

**Results:**

The diversity indexes showed significant differences in the microbial community among the primary HSP groups, the recurrent HSP group and healthy controls. The abundance of *Escherichia-Shigella* in the recurrent HSP group was significantly higher than that in the primary HSP group, and the constructed ROC curve had an AUC value of 0.750. According to the Spearman correlation analysis, the abundance of *Bacteroides* was positively associated with the serum IgG level in children with HSP, while the abundance of *Lachnoclostridium* was negatively correlated with the complement component 3 (C3). The diversity indexes of gut microbiota in the HSP group with abdominal symptoms were higher than those in the HSP group without GI involvement, and also higher than those in the healthy control group. In the HSP group with GI involvement, the abundance of *Faecalibacterium* was decreased, while the abundance of *Streptococcus* and *Fusobacteria* was increased, compared to the HSP group without GI involvement.

**Conclusions:**

The gut microbiota of children with HSP was different from that of healthy children. The genus *Escherichia-Shigella* has a diagnostic value for HSP recurrence. *Bacteroides* and *Lachnoclostridium* may affect IgG and complement C3 levels in children with HSP. Abdominal symptoms in HSP children were related to gut microbiota (*Streptococcus* and butyric acid-producing bacteria).

## Introduction

Henoch-Schonlein Purpura (HSP), also known as immunoglobulin A vasculitis, is an autoimmune disease commonly occurring in children, affecting 3–27 per 100,000 children (Oni and Sampath, 2019). The clinical manifestations of HSP include non-thrombocytopenic purpura, abdominal discomfort, joint pain, and renal involvement (Ozen et al., 2010). The pathogenesis of HSP is complex and remains elusive. It is generally accepted that both genetic and environmental factors contribute to HSP pathogenesis (Heineke et al., 2017). In genetically predisposed individuals, HSP can be initiated by various environmental factors, including bacterial, viral, or parasitic infection, allergy, and use of drugs and vaccines (Da et al., 2016; Hwang et al., 2018). Among them, gut infection by bacteria such as *Helicobacter pylori* may trigger HSP development through activation of complements, which can be considered a significant factor involved in HSP pathogenesis (Xiong and Mao, 2016). Moreover, accumulating evidence suggests that alterations in gut microbiota composition may play an important role in the pathogenesis of various immune-mediated diseases, such as inflammatory bowel disease (Franzosa et al., 2019), systemic lupus erythematosus (Li et al., 2020), and IgA nephropathy (Hu et al., 2020). Therefore, disturbance of the gut bacteria may be associated with the pathogenesis of HSP.

A previous study reported differences in gut microbiota composition and abundance between patients with HSP and healthy individuals (Wang et al., 2018). However, correlations between the gut microbiota, different disease stages of primary and recurrent HSP, and different clinical manifestations remain undefined. The present study aimed to identify specific microbiota in patients with HSP and analyze the relationship between the microbiota and different clinical states of HSP through high-throughput sequencing of 16S rRNA V3-V4 variable regions to determine strategies to reduce the risk of recurrence of HSP.

## Materials and Methods

### Study Subjects

Included in this study were 68 patients with HSP diagnosed between December 2018 and December 2019 at the Department of Pediatrics of Dongfang Hospital affiliated to Xiamen University, the Fujian Provincial Maternity and Children’s Hospital, Fujian Provincial Hospital, and Fujian Medical University Union Hospital (Fuzhou, China). The inclusion criteria for HSP patients were as follows: 1) patients younger than 18 years; 2) diagnosis of HSP requiring the presence of palpable purpura with at least one of the following four characteristics: abdominal pain, arthritis or arthralgia, renal involvement, and biopsy revealing leukocytoclastic vasculitis with predominant IgA deposits or proliferative glomerulonephritis with predominant IgA deposits; 3) recurrence of HSP, which was defined as patients with a pre-existing diagnosis of HSP but having no symptoms for at least 4 weeks who presented symptoms again (Calvo-Rio et al., 2016); 4) informed consent obtained from the participating patients. The exclusion criteria were patients who used any type of antibiotics or immunosuppressors within 2 weeks before fecal collection (Gondalia et al., 2012; MacPherson et al., 2018). The inclusion criteria for healthy children: 1) age younger than 18 years; 2) no history of allergic rhinitis, allergic asthma, allergic cough and other allergic diseases; 3) no antibiotics, proton pump inhibitors, immunosuppressants, chemotherapy drugs, or radiotherapy drugs, used within 2 weeks before specimen collection. This study was approved by the Department of Pediatrics of Dongfang Hospital, affiliated with Xiamen University (no. 2019-009).

### Fecal Sample Collection and DNA Extraction

Both patients and healthy controls were taught how to collect the middle part of their stools in disposable sterile containers in a clean manner wearing disposable gloves on both hands. The freshly collected stool samples were immediately stored at -80°C before sequencing was performed by Fujian Xilong Biotech Co., Ltd., Fuzhou, China.

Total genomic DNA was extracted from each sample using a HiPure Stool DNA Kit (Meiji Co., Ltd., Guangzhou, China) according to manufacturer’s instructions. The DNA concentration was diluted to 10 ng/µL with sterile water for PCR amplification.

### 16S rRNA Gene Sequencing

To analyze the microbial diversity in the stool samples, the bacterial 16S rRNA gene was amplified using primers specific for the V3-V4 hypervariable regions (515F: 5′-ACTCCTACGGGAGGCAGCAG-3′ and 806R: 5′-GGACTACHVGGGEATWTCTAAT-3′) in a thermocycler PCR system (Fujian Xilong Biotech Co., Ltd., Fuzhou, China). After two-step purification and addition of the adaptor, the libraries were used for sequencing on a MiSeq PE300 (Xilong Bio Company., Fuzhou, China).

### Bioinformatics Analysis

Using QIIME software, α-diversity, including ACE, Chao, Sobs, and Simpson indexes were calculated to reflect the abundance and homogeneity of the gut microbiota. β-diversity was estimated through principal coordinate analysis (PCoA) and analysis of similarities (ANOSIM). PCoA was performed to determine whether there are significant differences in the microbial community between primary HSP, recurrent HSP, and healthy control groups. Inter-group differences were compared through ANOSIM. Using linear discriminant analysis (LDA) effect size (LEfSe), inter-group differences in differential abundance were analyzed at the phylum, class, order, family, genus, and species levels, and the significance between the three groups was assessed through LDA, which was defined as *P* < 0.05.

### Receiver Operating Characteristic Analysis

Receiver operating characteristic (ROC) analysis, a tool used to describe the discrimination accuracy of a diagnostic test or prediction model, was performed using the pROC package in R (version 3.6.3), and the area under the curve (AUC) was used to assess the ROC effect.

### Statistical Methods

Statistical analysis was performed using SPSS 20.0 software. Descriptive statistics are expressed as the mean ± standard deviation (SD), median, or frequency. Comparison of continuous variables between two groups was performed using the Student’s *t*-test or Wilcoxon rank-sum test. For a comparison of more than two groups, a one-way analysis of variance was performed. Correlation analysis was conducted using Pearson’s product-moment coefficient. Significance was accepted at *P* < 0.05.

## Results

### Subject Characteristics

Altogether, 34 patients with HSP (18 with primary HSP and 16 with recurrent HSP) and 23 healthy controls were recruited in this study (**Table 1**). Thirty-four initially recruited patients with HSP were excluded from the study because of antibiotic or immunosuppressant use.

**Table 1:** Characteristics of study participants.

	P-HSP	R-HSP	HC	P value
Subjects (n)	18	16	23	
Age(years, mean ± SD)	6.38 ± 2.47	8.98 ± 3.31	7.26 ± 3.29	.058
Gender (n)				.844
Boy	11	6	13	
Girl	7	10	10	
Clinical presentation (n)				
Skin involvement	8	4	/	
Skin, GI involvement	2	0	/	
Skin, musculoskeletal involvement	2	0	/	
Skin, renal involvement	2	11	/	
Skin, GI and musculoskeletal involvement	2	0	/	
Skin, GI and renal involvement Skin, musculoskeletalt and renal involvement	2 0	0 1	/ /	
Skin, GI, musculoskeletal and renal involvement	0	0	/	

Results are showed as the mean ± SD and frequency.

SD, standard deviation; P-HSP, primary HSP; R-HSP, recurrent HSP; HC, healthy controls; GI, gastrointestinal.

### Diversity of the Bacterial Community in Primary HSP, Recurrent HSP, and Healthy Control Groups

**Figure 1:**
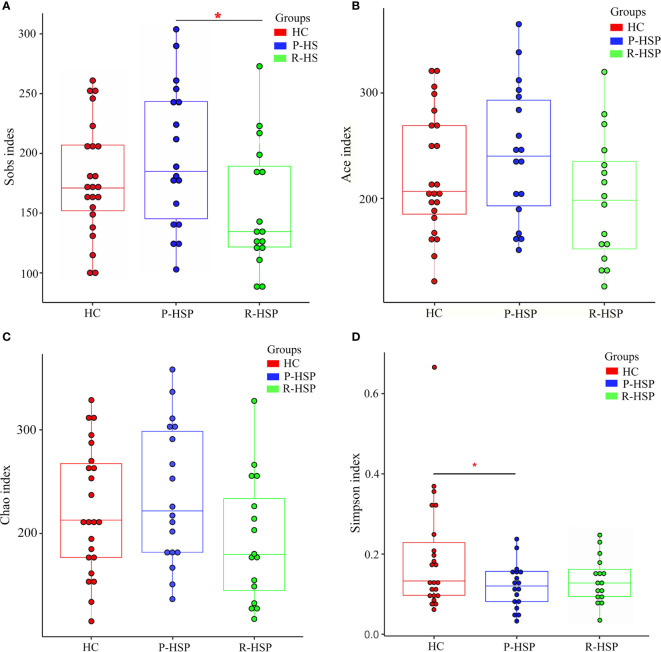
Comparison of α-diversity indexes between patients with primary HSP and recurrent HSP and healthy controls in terms of species richness and adversity. The Y-axes stand for the scores of the α-diversity index, and the X-axes show the phenotypic categories. The box depicts the interquartile range between the first (25^th^ percentile) and third (75^th^) percentile, and the line inside the box denotes the median. **(A)** The Sobs index displays higher species richness in patients with primary HSP than in patients with recurrent HSP and healthy controls, but the difference was significant only when compared with the species richness in patients with recurrent HSP (*P* < 0.05). **(B)** There was no significant difference in ACE indexes between the three groups (primary HSP *vs*. healthy controls *P* > 0.05; recurrent HSP *vs*. healthy controls *P* > 0.05; primary HSP *vs*. recurrent HSP *P* > 0.05). **(C)** Chao index was the lowest in the recurrent HSP group, but the difference was not statistically significant (primary HSP *vs*. healthy controls *P* > 0.05; recurrent HSP *vs*. healthy controls *P* > 0.05; primary HSP *vs*. recurrent HSP *P* > 0.05). **(D)** The Simpson diversity index in the primary HSP group was lower than that in the healthy control group (*P* < 0.05) and recurrent HSP group (*P >* 0.05), but the difference was not statistically significant. *P < 0.05.

**Figure 2:**
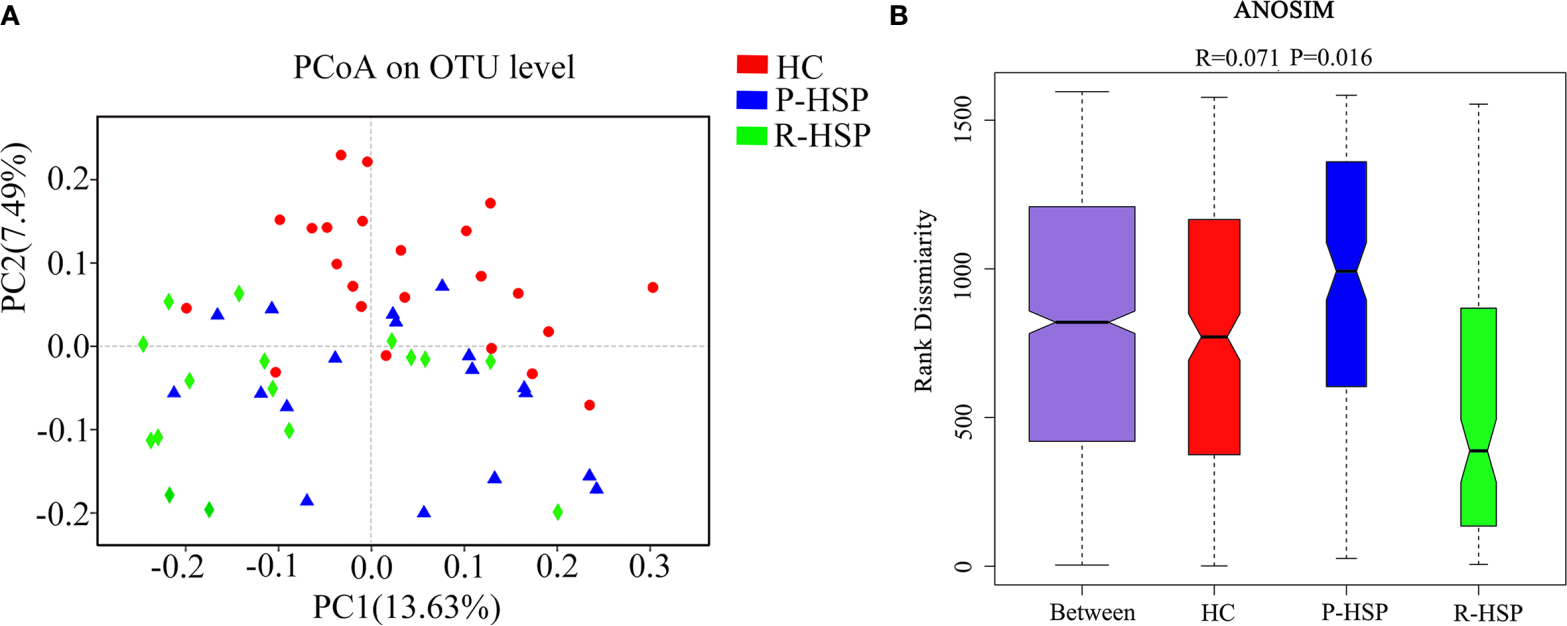
Comparison of β-diversity indexes between patients with primary HSP and recurrent HSP and healthy controls. **(A)** PCoA analysis was based on unweighted UniFrac distance for bacterial communities. The closer the samples, the more similar the groups were. PCoA analysis showed the differences in the gut microbial composition between the three groups. **(B)** Analysis of similarities (ANOSIM) was based on the unweighted UniFrac distance between the groups. The ANOSIM that generated R test statistics ranging from −1 to 1 was used to measure clustering of the samples patient-wise. A positive R-value indicates greater within-group similarity than between-group similarity, with greater magnitudes of the R-value suggesting stronger sample clustering. An R-value of 0 indicates no clustering of the samples, whereas a negative R-value suggests greater between-group resemblance than within-group similarity. The ANOSIM demonstrated clear differences in the gut microbial composition between the two HSP groups and the healthy control group (R = 0.071, *P* = 0.016).

A total of 3,385,069 sequences and 1,424,697,702 bases were obtained from the 57 samples, including 34 HSP patients and 23 healthy controls, with an average of 420.6501694 average lengths per sample. To determine α-diversity, we calculated the mean ACE, Chao, Sobs, and Simpson indexes (**Figure 1**). The results showed no significant difference in ACE and Chao indexes, and a significant increase in Sob index in the primary HSP group compared with those in the recurrent HSP group (*P* < 0.05). Next, we assessed the bacterial community diversity and found that the Simpson index in patients with primary HSP was lower than in healthy controls (*P* < 0.05). In β-diversity analysis, we found that PCoA could discriminate against the healthy control samples from primary and recurrent HSP samples (**Figure 2A**). The ANOSIM test further confirmed the significant difference in the microbial community structure among the three groups (**Figure 2B**). To investigate whether changes in the gut microbiome in the two HSP groups were associated with other variables such as age, gender, and special diets, we performed multivariate analysis. As both the HSP patients and healthy controls were from Fujian, China, there should be no significant differences in dietary habits. Our results indicated that age, gender, and diet did not significantly impact the gut microbiota diversity (*P* > 0.05) (**Table 1**).

### Differences in the Bacterial Community Structure Between Primary HSP, Recurrent HSP, and Healthy Control Groups

**Figure 3:**
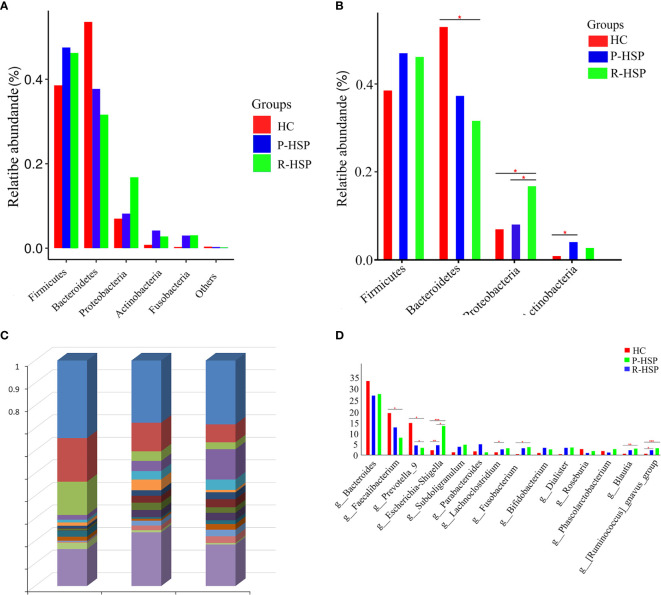
Histogram of relative abundance (%) of intestinal flora at different taxonomic levels in children with primary and recurrent HSP and healthy controls. **(A)** Relative abundance of each phylum in two HSP groups and a healthy control group. Firmicutes, Bacteroidetes, Proteobacteria, and Actinobacteria phyla encompassed most bacteria in the fecal samples of patients with HSP and healthy controls. The sum of the percentages reached 90% in each group. **(B)** Significance of abundance of the top four community bacteria at the phylum level. We observed that Firmicutes and Bacteroidetes constituted the most abundant phyla in both HSP and healthy control groups, but with no significant difference between the primary HSP and healthy control groups. No significant difference was observed in the microbial composition at the Firmicutes and Actinobacteria level between primary and recurrent HSP groups or between recurrent HSP and healthy control groups. **(C)** The microbiome composition in all three groups at the genus level. The figure shows the top 15 species in each group based on their relative abundances. *Bacteroides, Faecalibacterium*, *Parabacteroides*, *Escherichia-Shigella*, and *Prevotella_9* were the top five genera in the primary HSP group, and *Bacteroides, Escherichia-Shigella*, *Faecalibacterium*, *Subdoligranulum*, and *Fusobacterium* were the top five genera in the recurrent HSP group. In the healthy controls group, the dominant microbiota was *Bacteroides*, *Faecalibacterium*, *Prevotella_9*, *Escherichia-Shigella*, and *Roseburia*. **(D)** Significance of abundance of the 14 community bacteria at the genus level. The *Escherichia-Shigella*, *Blautia*, and *Ruminococcus_gnavus* were more abundant in recurrent HSP group than in primary HSP and healthy control groups, while *Faecalibacterium* and *Prevotella_9* were more abundant in the healthy control group compared with that in HSP groups. *P < 0.05, **P < 0.01, ***P < 0.001.

**Figure 4:**
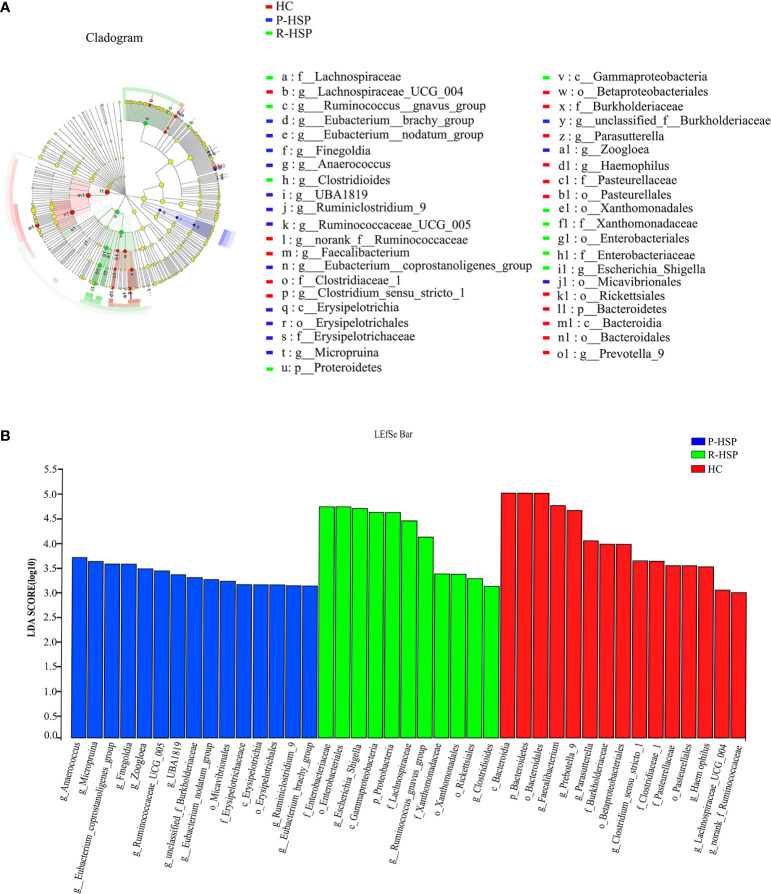
Differentially abundant bacterial taxa associated with patients with HSP and healthy controls according to LEfSe analysis. **(A)** Cladogram showing the phylogenetic distribution of the bacterial lineages in the three groups. The phylum, class, order, family, and genus levels are listed in order from the inside to the outside of the cladogram, and different-colored regions represent different constituents: healthy control group is shown in red, primary HSP in blue, and recurrent HSP in green, whereas the yellow circles represent taxa with no significant differences between the three groups. The picture shows that Bacteroidetes (from phylum to genus) are prominent in the healthy control group, Proteroidetes (from phylum to genus) are significantly enriched in the recurrent HSP group, and Erysipelotrichia (from class to genus) are enriched in the primary HSP group. **(B)** Horizontal bars represent the effect size for each taxon. The length of the bar represents the log10 transformed LDA score as indicated by vertical dotted lines. The threshold on the logarithmic LDA score for discriminative features is set to 4.0. LDA scores show significant bacterial differences between the three groups. The healthy control group is characterized by a preponderance of *Faecalibacterium* and *Prevotella_9* (LDA score [log10] > 4.5) and recurrent HSP is characterized by a preponderance of *Escherichia-Shigella* (LDA score [log10] > 4.5). p, phylum; c, class; o, order; f, family; g, genus.

As shown in **Figure 3** and **Table 2**, Firmicutes, Bacteroidetes, Proteobacteria, and Actinobacteria were the dominant phyla in all samples (**Figure 3A**). The bar chart shows that the abundance of Proteobacteria had an increasing tendency in recurrent HSP group compared with that in primary HSP (*P* < 0.05) and healthy control groups (*P* < 0.05). In contrast, the proportion of Bacteroidetes in the recurrent HSP group was significantly lower than that in the healthy control group (*P* < 0.05) (**Figure 3B**). At the genus level, we identified 12 different genera in the two HSP groups, where the mean abundance was over 1%, as shown by the Kruskal-Wilcoxon test (**Figure 3C**). The 12 genera identified were characterized by an increase in the abundance of the genera *Escherichia-Shigella*, *Blautia*, *Subdoligranulum*, *Lachnoclostridium*, *Fusobacterium*, and *Ruminococcus_gnavus* and a decrease in the abundance of the genera *Prevotella_9* and *Faecalibacterium* in the three groups (**Figure 3D**). To identify bacterial taxa that are more specifically associated with HSP, the gut microbiota between patients with HSP and healthy controls was compared using LEfSe analysis (**Figure 4A**). At the phylum level, the relative abundance of Bacteroidetes and Proteobacteria was enriched in healthy and recurrent HSP groups, respectively, while no phylum was enriched in the primary HSP group. At the genus level, seven, eleven, and three genera were enriched in healthy, primary HSP, and recurrent HSP groups, respectively. LDA showed that when the LDA threshold was increased to 4.5, the genus *Escherichia-Shigella* was enriched at the genus level in the recurrent HSP group (**Figure 4B**). These results suggest that certain genera of the gut microbiota may be potential biomarkers or even potentially beneficial microbiota for preventing HSP recurrence.

**Table 2:** P-value statistics of differences among intestinal microbial groups at different taxonomic levels.

	HC *vs*. P-HSP	P-HSP *vs*. R-HSP	HC *vs*. R-HSP
Phyla			
Bacteroidetes	0.0535	0.6047	0.01151
Proteobacteria	1	0.04355	0.01997
Firmicutes	0.3118	0.6915	0.4842
Actinobacteria	0.04164	0.2477	0.8977
Genera			
Bacteroides	0.3443	0.08766	0.4158
Faecalibacterium	0.09528	0.1841	0.01352
Prevotella_9	0.4415	0.04654	0.01236
Escherichia-Shigella	0.005783	0.01363	0.000194
Subdoligranulum	0.5453	0.545	0.8405
Parabacteroides	0.2531	0.06468	0.3456
Lachnoclostridium	0.03445	0.9587	0.09206
Fusobacterium	0.08863	0.7671	0.02929
Bifidobacterium	0.2218	0.162	0.5021
Dialister	0.5912	0.831	0.9767
Roseburia	0.06387	0.8349	0.07154
Phascolarctobacterium	0.5595	0.5525	0.8624
Blautia	0.1243	0.3789	0.001948
[Ruminococcus]_gnavus_group	0.01036	0.1897	0.000153

**Figure 5:**
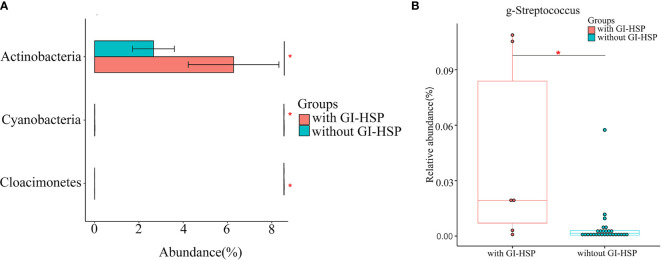
Relative abundance (%) and taxonomic differences of gut microbiota in HSP patients with and without abdominal pain. **(A)** The relative abundance of the phylum *Actinobacteria* in patients without GI involvement was significantly lower than that in patients with GI involvement. **(B)** The relative abundance of *Streptococcus* in each sample. Box and whisker plots show that *Streptococcus* relative abundance was significantly increased in patients with GI involvement compared with those in patients without GI involvement. *P < 0.05.

To detect the specific bacteria associated with HSP clinical presentation, we next examined whether the gut microbial profile was different between HSP patients with and without GI involvement. Compared with the patients without GI involvement, the abundance of Actinobacteria was significantly higher at the phylum level (**Figure 5A**), and that of *Streptococcus* was enriched at the genus level (*P* < 0.05) (**Figure 5B**) in patients with GI involvement.

### ROC Curve Analysis

**Figure 6:**
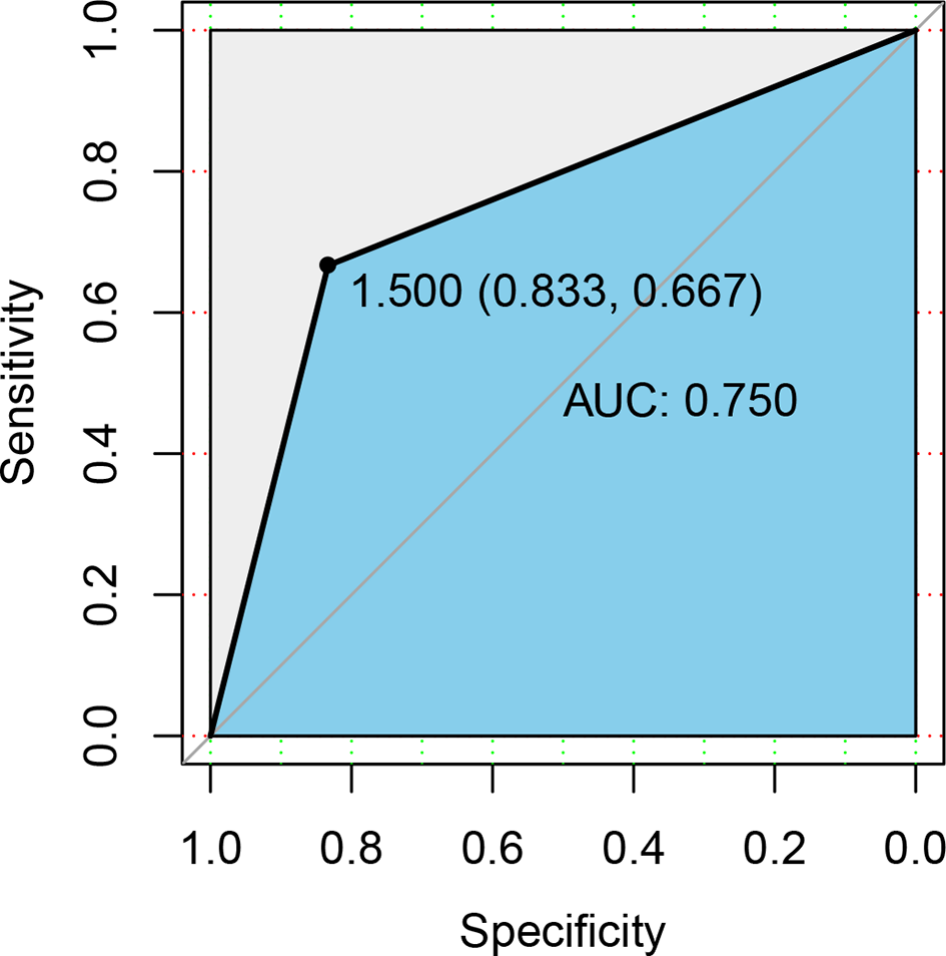
ROC curve differentiation of patients with HSP from healthy groups according to the relative abundance at the genus level. We used ROC analysis to assess how well specific genera of the gut microbiota could discriminate between individuals who experienced disease recurrences and those who did not. The yellow line is the mean ROC curve, and the average area under the ROC curves (AUC) is 0.750, indicating good performance.

The above results demonstrated significant differences in intestinal microbiota between primary HSP and recurrent HSP groups. Subsequently, we sought to identify biomarkers that could distinguish the two groups. We used the Random Forest algorithm of machine learning to select the top five genera (*Prevotella_9, Faecalibacterium, Blautia, Escherichia-Shigella*, and *Ruminococcus gnavus*) whose abundance was significantly different between the two groups, and then used these microbiota to construct the work curve of ROC (**Figure 6**). The AUC of the ROC curve was 0.750, showing that the model could be used to distinguish the two groups. Meanwhile, the model specificity and sensitivity were 83.3%, and 66.7%, respectively, showing high diagnostic efficiency, indicating that the gut microbiota is a strong predictor of recurrence.

### Correlation Analysis of Clinical Indicators and Bacterial Flora

**Figure 7:**
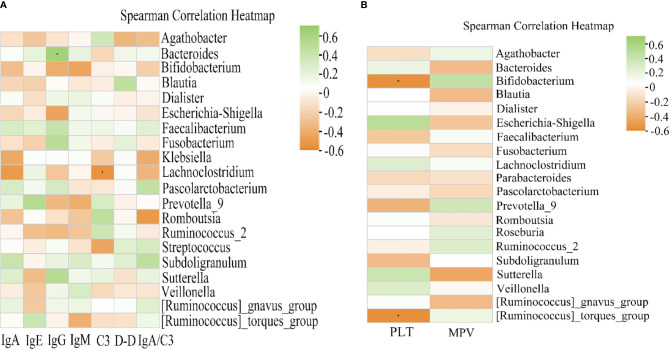
Heat map of Spearman correlation analysis between the gut microbiota and clinical indices of HSP. **(A)** Correlation between the relative abundance in HSP groups and the clinical indices. IgG exhibited a significant positive correlation with the genus *Bacteroides* (r = 0.5347, *P* < 0.05). C3 was negatively correlated with *Lachnoclostridium* (r = -0.5523, *P* < 0.05). **(B)** Correlation between the relative abundance of gut microbiota and the clinical indices in recurrent HSP. Spearman analysis showed a strong negative correlation between the platelet count and *Bifidobacterium* (*r* = -0.5533, *P <* 0.05) and *Ruminococcus_torques_group* (r = -0.5659, *P* < 0.05). Green and orange represent positive and negative correlations, respectively. *P < 0.05.

Given that there are significant immune disorders including elevated IgA and IgE levels in patients with HSP, we conducted Spearman correlation analysis to determine the potential correlation between the gut microbiota and HSP-related indexes including IgA, IgE, IgG, IgM, C3, D-dimer, and IgA/C3 (**Figure 7A**). The results showed that *Bacteroides* was positively associated with IgG (r = 0.5347, *P* < 0.05), and the abundance of *Lachnoclostridium* was negatively correlated with C3 (r = -0.5523, *P* < 0.05).

Moreover, the prognosis of HSP is generally good, but recurrence is common in pediatric patients. Various predictors, including platelet counts and mean platelet volume, may be early clues for predisposition to recurrent vascular inflammation (Ekinci et al., 2019). The Spearman correlation analysis showed that the abundance of *Bifidobacterium* (r = -0.5533, *P* < 0.05) and [*Ruminococcus]_torques_group* (r = -0.5659, *P* < 0.05) had a significant negative correlation with the platelet count, while mean platelet volume was not associated with intestinal flora abundance (**Figure 7B**).

## Discussion

Although the intestinal flora is closely related to immune-mediated diseases, the gut microbiota profile in different stages of HSP patients has not been previously characterized. In this study, we found that the gut microbiota of patients with HSP was in a state of dysbiosis, fecal microbiota underwent changes in different stages of HSP, and some clinical indexes may be related to the gut microbiota. In summary, specific changes in the microbiota composition may cause phenotypic differences in children with HSP.

In the present study, we found that the abundance of *Proteobacteria* was significantly increased in patients with HSP, and this increasing tendency was associated with relapse frequency. Proteobacteria is one of the most abundant phyla in the human gut microbiota, comprising several pathogenic species (Rizzatti et al., 2017). A previous study demonstrated that an increased abundance of Proteobacteria is a potential microbial diagnostic marker of dysbiosis (Shin et al., 2015). For example, hyperproliferative Proteobacteria is found in the feces of patients with Crohn’s disease (Kiernan et al., 2019), autism spectrum disorders (Coretti et al., 2018), systemic lupus erythematosus (He et al., 2016), and food sensitization (Chen et al., 2016). Carvalho et al. reported that Protebacteria can enhance the susceptibility to intestinal damage in patients with a dysregulated innate immune system (Carvalho et al., 2012). All these findings support the presence of gut microbiota dysbiosis in patients with HSP.

The alpha diversity indexes in the present study demonstrated that the diversity of the bacterial communities was lower in patients with primary HSP than in the health group, and that the abundance of *Dialister* in the gut of patients with HSP was significantly higher than that in healthy controls, which are discrepant with the findings of Wang et al. (2018). In addition, we observed that *Enterococcus* and *Escherichia-Shigella* were significantly enriched, and *Roseburia* and *Parasutterella* were significantly decreased in patients with HSP, which is consistent with the findings of Wang et al. (2018). Discrepancies among high-throughput 16S rDNA sequencing studies are common and can result from several reasons, such as sample source, differences in the experimental setting, different PCR primers, and statistical tests (Kang et al., 2013). The reasons underlying the discrepancies between our results and previous research may be partly related to differences in the inclusion criteria, disease stages, and diets. In our study, we defined HSP as the presence of non-thrombocytopenic palpable purpura with at least one systemic involvement, whereas in previous studies the inclusion criteria included at least two systemic involvements. In addition, the authors did not discriminate between primary HSP and recurrent HSP.

In this study, we determined the potential biomarkers of different groups through LEfSe. The relative abundance of *Escherichia-Shigella*, *Ruminococcus gnavus*, and *Clostridioides* was significantly higher in the recurrent HSP group than in the other two groups. Among them, *Escherichia-Shigella*, in particular, contributed to the major difference between the three groups according to the results of LDA. The genus *Escherichia-Shigella*, a member of the family E*nterobacteriaceae*, comprises predominantly facultative anaerobic gram-negative bacteria that colonize the intestinal tract and help maintain intestinal homeostasis under normal conditions. However, the destruction of the intestinal microbiota due to host-mediated inflammation may promote the overgrowth of *Enterobacteriaceae* (Lupp et al., 2007). Compared with healthy children, *Escherichia-Shigella* was significantly increased in infants with eczema (Zheng et al., 2016), food allergy (Shen et al., 2019), and inflammatory bowel disease (Chen et al., 2014; Mirsepasi-Lauridsen et al., 2019). Additionally, Kau et al. reported that the mucosal immunity is interrupted by the *Enterobacteriaceae* and intestinal mucosal IgA responses under malnutrition (Kau et al., 2015). Garrett et al. also showed that B2 *Escherichia coli* is translocated to the coelenteron through intestinal epithelia, impairing intestinal permeability, altering immune homeostasis, and facilitating the development of deregulated immune-mediated diseases (Garrett, 2019). In addition, we used ROC analysis to further determine whether *Escherichia-Shigella* can be used as a potential biomarker to predict HSP recurrence. It was found that *Escherichia–Shigella* offered high classification accuracy, as evidenced by an AUC of 0.750 (**Figure 6A**). These results indicate that increased *Escherichia–Shigella* abundance is closely associated with HSP recurrence, and that overgrowth of this genus may lead to immune imbalance, thus promoting HSP recurrence. Notably, human ingestion of *Lactobacillus paracasei subsp*. *Paracasei LC01* significantly decreases *Escherichia-Shigella* abundance, suggesting that probiotics or probiotic foods may reduce the relative abundance of *Escherichia-Shigella* in the intestinal tract of patients with HSP and effectively reduce the risk of disease recurrence (Zhang et al., 2013).

GI involvement is common in about 50–85% of patients with HSP who have abdominal pain, vomiting, diarrhea, and bloody stool as the main abdominal symptoms. A previous study revealed that the deposition of IgA immune complexes in small vessel walls could activate the alternate complement pathway, leading to neutrophilic accumulation (Han et al., 2019). This vascular inflammation causes extravasation of red blood cells and fibrin deposition in interstitial tissues, leading to abdominal symptoms. We used 16SrRNA sequencing to identify gut microbiota composition in HSP patients with and without GI involvement and found that abundance of the genus *Streptococcus* was significantly increased in patients with GI involvement (**Figure 5B**). *Streptococcus* is also a common commensal and opportunistic pathogen. However, it can cause infections when gut microbiota dysbiosis occurs. Kieser et al. reported that abundance of the genus *Streptococcus* is increased in patients with acute diarrhea (Kieser et al., 2018). In addition, the abundance of *Streptococcus* is significantly increased in children with primary sclerosing cholangitis, but the specific mechanism is unknown (Hov and Karlsen, 2017). Another study reported that highly abundant *Streptococcus* enters the blood circulation, where it triggers macrophage activation through TLR pathways, which not only induces inflammatory responses but activates mucosal immunity, leading to inflammation in tissues and visceral organs (Boer et al., 2019). This is consistent with the previous finding that macrophage activation is enhanced in children with nephritis in HSP (Xu et al., 2017). In patients with HSP, the serum level of galactose-deficient IgA1 was significantly elevated, and this terminal glycan of IgA1 was better recognized by autoantibodies, causing IgA aggregation and generation of macromolecular immune complexes. Studies have shown that when *Streptococcal* infection occurs, the N-acetylgalactosamine structure on its surface forms an immune complex with IgAI, which can deposit in the GI tract (Goyette-Desjardins et al., 2018). This may explain why *Streptococcus* is common in patients with HSP and is frequently characterized by abdominal symptoms.

Complement activation of the alternative pathway by IgA and IgM aggregates has been thought to play an important role in the pathogenesis of HSP. C3 is an important complement, and some studies reported that IgG combined with immunoglobulin deposition may induce complement activation (Evans et al., 1973; Park et al., 2013).We therefore performed Spearman correlation analysis to determine the potential correlation between gut microbiota and HSP-related indexes and found that IgG was positively associated with *Bacteroides*, and *Lachnoclostridium* was negatively correlated with C3. *Bacteroides* are the most abundant gram-negative bacterial strains within the human intestinal tract. Notably, this genus can break down diet and host-derived polysaccharides using rich glycan utilization systems, and then produce short-chain fatty acids (SCFAs). Yanagibashi et al. showed that *Bacteroides*-mediated acidification promotes IgA production in the large intestine due to an increase in the number of IgA^+^ B cells (Yanagibashi et al., 2013). Moreover, several studies have shown that SCFAs can effectively increase B cell metabolism and promote increased IgA and IgG levels in the blood (Kim et al., 2016; Zhang et al., 2019). In the present study, we found that the relative abundance of *Bacteroides* in the primary HSP group was lower than that in the recurrent HSP group, but the difference was not statistically significant. Therefore, Bacteroidetes may increase IgA and IgG deposition through SCFAs and exacerbate HSP symptoms when gut microbiota dysbiosis occurs. *Lachnoclostridium* is a newly defined genus in the class *Clostridium* and has been detected in human intestinal flora in recent years (Liang et al., 2020). However, no studies on *Lachnoclostridium* and complement have been reported so far, and the relationship between the microbiota and HSP should be further examined.

We further found that *Bifidobacterium* and *Ruminococcus_torques_group* were negatively correlated with platelet count. Platelets play an important role in acute and chronic inflammation, including the release of their pro-inflammatory mediators and close interaction with leukocytes and endothelial cells (Asarat et al., 2016). As HSP is the most common systemic vasculitis, overproduction of pro-inflammatory cytokines such as TNF-a, IL-6, and IL-8 may be involved in the disease pathogenesis (Park et al., 2013). Lin et al. showed that thrombocytosis in patients with HSP is a type of inflammatory reactive thrombocytosis and that platelets may be related to IL-6 (Lin et al., 2006). *Bifidobacteria* are commensal microorganisms of the human GI tract and are thought to play pivotal roles in maintaining human health. Moreover, studies have shown that *Bifidobacteria* can significantly inhibit TNF-α and IL-6 levels (Lu et al., 2019; Nie et al., 2020). In the present study, we found that the abundance of *Bifidobacterium* in patients with primary HSP was higher than in patients with recurrent HSP but the difference was not statistically significant. Therefore, *Bifidobacteria* may reduce HSP recurrence by reducing inflammatory factors. *Ruminococcus_torques_group* are well-known butyrate-producing bacteria of the phylum Firmicutes. As mentioned above, SCFAs are associated with the downregulated excretion of multiple pro-inflammatory cytokines, such as IL-2, IL-6, and IFN-α (Kyventidis et al., 2015; Asarat et al., 2016). These cytokines are the main mediators of the inflammatory response and play crucial roles in HSP pathogenesis. Luu et al. showed that SCFAs suppress inflammation by inducing the expression of IL-10 in effector T cells (Luu et al., 2019). In contrast, we found that *Ruminococcus_torques_group* was present in higher abundance in recurrent HSP group *vs*. primary HSP and healthy control groups. Wang et al. also showed that the relative abundance of *Ruminococcus_torques_group* is increased in children with ASD and functional GI disorders, though the exact mechanism is unclear (Wang et al., 2013). Png et al. also reported an increased abundance of *Ruminococcus_torques_group* in adults with IBD, whose enhanced growth rate may be triggered by mucin. The association between *Ruminococcus_torques_group* and platelets is unclear and requires further study (Png et al., 2010).

Our study has some limitations. First, only samples from 57 subjects were included for analysis, and thus larger cohorts are required to verify our findings and conclusions. Second, diets and geographical locations can influence the microbiota composition. As all samples in this study were from Fujian Province, the gut microbiota effect in patients with HSP from other provinces should be evaluated in future studies. Finally, recent studies indicate that microbiota is crucial in the development of autoimmune diseases (Bibbo et al., 2020). Some HSP patients combined with other autoimmune diseases, whether there is a difference in the composition of the gut microbiota between HSP patients with and without poly-autoimmune disease has not been clarified. Further study is necessary.

In conclusion, our study demonstrated that the gut microbiota undergoes significant alterations in patients with HSP, and changes in the abundance of specific gut microbes may be useful for early diagnosis of HSP, leading to a reduction in disease recurrence. Microbial treatment may be useful as an ecological therapy in the treatment of HSP.

## Data Availability Statement

The original contributions presented in the study are publicly available. This data can be found in NCBI under accession number PRJNA687642.

## Ethics Statement

The studies involving human participants were reviewed and approved by Department of Pediatrics of Dongfang Hospital, affiliated with Xiamen University (no. 2019-009). Written informed consent to participate in this study was provided by the participants’ legal guardian/next of kin.

## Author Contributions

All authors listed have made a substantial, direct, and intellectual contribution to the work, and approved it for publication.

## Funding

This work was supported in part by the National Natural Science Foundation of China (grant number 81300583); Science and Technology Innovation Project of Fujian Province (2019Y9043); Key Project of Social Development of Fujian Province of China (grant number 2019Y0069).

## Conflict of Interest

The authors declare that the research was conducted in the absence of any commercial or financial relationships that could be construed as a potential conflict of interest.
